# Assessment of Left Ventricular Systolic Function by Cardiovascular Magnetic Resonance Compressed Sensing Real-Time Cine Imaging Combined With Area-Length Method in Normal Sinus Rhythm and Atrial Fibrillation

**DOI:** 10.3389/fcvm.2022.896816

**Published:** 2022-05-27

**Authors:** Gang Yin, Chen Cui, Jing An, Kankan Zhao, Kai Yang, Shuang Li, Xinling Yang, Jiaxin Wang, Zhixiang Dong, Shiqin Yu, Jian He, Xiuyu Chen, Minjie Lu, Shihua Zhao

**Affiliations:** ^1^MR Center, Stata Key Laboratory of Cardiovascular Disease, National Center for Cardiovascular Diseases of China, Chinese Academy of Medical Sciences and Peking Union Medical College, Fuwai Hospital, Beijing, China; ^2^Siemens Shenzhen Magnetic Resonance Ltd., Siemens MRI Center, Shenzhen, China; ^3^Paul C. Lauterbur Research Center for Biomedical Imaging, Shenzhen Institutes of Advanced Technology, Chinese Academy of Sciences, SZ University Town, Shenzhen, China

**Keywords:** cardiovascular magnetic resonance, compressed sensing real-time, area-length, normal sinus rhythm, atrial fibrillation

## Abstract

**Background:**

The most-commonly used multi-slice Simpson's method employed with routine two-dimensional segmented cine images makes it difficult to evaluate left ventricular (LV) volume and function due to endocardial border blurring and beat-to-beat variation during atrial fibrillation (AF) status.

**Objectives:**

To assess the feasibility of compressed sensing real-time (CSRT) cine imaging combined with an area-length method for quantification of LV systolic function in normal sinus rhythm (NSR) and AF.

**Methods:**

The CSRT cine sequence and routine segmented balanced Steady-State-Free-Precession cine sequence were performed in 71 patients with NSR (*n* = 36) or AF (*n* = 35). Image quality and edge sharpness for both sequences were assessed. The LV functional measurements in patients with NSR included end-diastolic volume (EDV), end-systolic volume (ESV), stroke volume (SV), ejection fraction (EF), cardiac output (CO), cardiac index (CI), and LV mass (LVM); all were assessed using segmented cine with Simpson's rule in short axis (SegSA_Simpson, as a reference standard) and area-length (AL) method in the two chamber (Seg2CH_AL) or four chamber (Seg4CH_AL) and CSRT cine with AL method in the two chamber (CSRT2CH_AL) or four chamber (CSRT4CH_AL). Finally, the mean, maximum, and minimum values of each LV functional parameter [EDV/ESV/SV/EF/CO/CI/LVM/heart rate (HR)] from 4~5 consecutive heartbeats were measured using CSRT2CH_AL in patients with AF.

**Results:**

In patients with NSR, measurements of EDV (*p* > 0.05), ESV (*p* > 0.05), SV (*p* > 0.05), EF (*p* > 0.05), and LVM (*p* > 0.05) assessed with CSRT2CH_AL did not differ significantly from those obtained with SegSA_Simpson. In patients with AF, CSRT image quality score (*p* < 0.001) and edge sharpness (*p* < 0.001) both were significantly higher than those obtained from segmented cine. The CSRT2CH_AL provided significantly different results among mean, maximum, and minimum values of each LV parameter from 4~5 consecutive heartbeats (all *p* < 0.001) with strong inter- and intra-observer agreement in AF.

**Conclusions:**

The CSRT cine sequence combined with two chamber area-length analysis accurately assessed LV systolic function in NSR. This approach is expected to permit the assessment of multiple parameters in consecutive heartbeats with good inter- and intra-observer reproducibility for beat-to-beat analysis of LV function in AF.

## Introduction

Cardiovascular magnetic resonance imaging (CMR) is well recognized as the “gold standard” for quantification of cardiac function (1–3). The Simpson method, which uses a stack of left ventricular (LV) short axis cine images derived from a conventional two-dimensional multi-breath-hold segmented balanced Steady-State-Free-Precession (bSSFP) sequence, has served as the standard approach for assessment of LV function in CMR (4, 5). Although the segmented bSSFP sequence provides high spatial and temporal image resolutions with high signal-to-noise ratio, this sequence cannot be used reliably if the patient has an irregular cardiac rhythm, especially in atrial fibrillation (AF), a condition that is considered an established and growing global epidemic (6). In AF patients, R-to-R (RR) interval irregularity and electrocardiograph (ECG) synchronization become challenging, resulting in blurring of segmented cine image quality or even nondiagnostic results. An algorithm named “Arrhythmia rejection” provided by some vendors can reject the data of too short or too long RR interval during MRI acquisition in slight arrhythmic patients. However, these algorithms often failed in AF due to the absence of any cardiac periodicity and the absence of appropriate threshold for arrhythmia rejection (7, 8). Furthermore, the parameters used to quantify systolic dysfunction in patients with normal sinus rhythm (NSR) also may be incorrect as a result of beat-to-beat variation in LV function (9, 10). Consequently, an accurate, feasible method for measuring LV volumes, ejection fraction, and cardiac output in a “one-stop-shop” CMR in AF patients remains highly desirable.

Real-time cine imaging combined with compressed sensing (CS) using a highly under-sampled k-space and a non-linear iterative reconstruction algorithm can provide spatiotemporal resolution in a range similar to that obtained with segmented cine (11–13). The area-length (AL) method has proved to be accurate in the assessment of symmetric LV volume and systolic function in animals and healthy volunteers (5, 14). The AL method also is well-accepted for quick assessment of LV volume and ejection fraction in echocardiography and LV angiography (15, 16).

Thus, the aim of this study was to evaluate the image quality of a compressed sensing real-time cine sequence and its feasibility for quantification of LV systolic function by the AL method in patients with NSR and AF.

## Materials and Methods

### Subjects

A total of 71 consecutive patients with a variety of cardiac diseases with NSR (36 patients, 24 male, 12 female; age range 16–71 years, mean ± standard deviation (SD), 44.2 ± 14.5 years) or sustained AF (35 patients, 25 male, 10 female; age range 35–81 years, mean ± SD, 56.9 ± 11.2 years) were enrolled in this study from May 2021 to September 2021. The persistence of AF was confirmed prior to the magnetic resonance imaging examination. The exclusion criteria for this study included cardiac implantable electronic device, claustrophobia, and other contraindications for CMR. Also excluded were patients who had an asymmetric left ventricle, which was defined as a ratio of septal-lateral to anterior-inferior diameter or of anterior-inferior to septal-lateral diameter exceeding 1.5, as determined in previous ultrasound or CMR short axis end diastole cine images (14); and patients who had non-uniform myocardial contraction, such as intraventricular block or regional wall motion abnormality (4). This prospective study was performed with institutional ethics committee approval, and all subjects provided written informed consent.

### CMR Protocol

All the CMR examinations were performed on a 3T MR scanner (MAGNETOM Skyra; Siemens Healthcare, Erlangen, Germany) using 18-channel surface phased array coils. Each subject underwent CMR scanning using a reference segmented (Seg) bSSFP cine sequence and a compressed sensing real-time (CSRT) bSSFP cine sequence during breath-holding; the two sequences were performed in random order. The CSRT sequence used in this study is a product sequence using prospective ECG gating, and its acquisition window is set to 6 seconds for covering several heartbeats. One two-chamber (2CH) slice, one four-chamber (4CH) slice, and a stack of eight to twelve short axis (SA) slices covering the whole LV from mitral valve to apex were acquired with the segmented sequence. Then, the same two long-axis slices and one short axis slice in the midventricular level (located at the same slice location as the reference sequence) were acquired with the CSRT sequence. The details of the imaging parameters for the two sequences are listed in Table 1.

**Table 1:** Imaging parameters of reference segmented cine and compressed sensing real-time cine.

	**Segmented cine**	**CSRT cine**
ECG mode	Retrospective	Prospective
Repetition time, ms	3.3	2.8
Echo time, ms	1.5	1.2
Field of view, mm^2^	360 ×297	380 ×300
Image matrix, pixels^2^	240 ×178	208 ×147
Flip angle, degrees	42–50	38–42
Spatial resolution, mm	1.7 ×1.5	2.0 ×1.8
Views per segment	12–13	15
Temporal resolution, ms	40–43	42
Slice thickness/gap, mm	8/2	8/2
Bandwidth, Hz/pixel	947	962
Cardiac phases	25	144
Dummy scan	1 heartbeat	0 heartbeat
Breath-hold	12 heartbeats/slice	6 second/slice
Acceleration factor	3	9.8

*CSRT, compressed sensing real-time; ECG, electrocardiograph*.

### Image Quality Assessment

For all qualitative and quantitative analyses, the first 10 phases (lasting about four hundred milliseconds) of the CSRT cine were discarded because of the lack of dummy scans. For both sequences, three location matched slices (2CH, 4CH, and one midventricular SA) were used to evaluate the image quality graded by two senior cardiovascular radiologists with more than 15 and 10 years of CMR experience respectively. Image quality scores were based on the border of myocardium and artifacts, and was graded using a 4-point Likert-type scale, as follows: 1 = nondiagnostic quality; 2 = fair quality, acceptable with some artifacts; 3 = good quality, few artifacts, diagnostic; and 4 = excellent quality, no artifacts. The two long axis and one midventricular SA slices were reviewed individually and an average image quality score was obtained as overall image quality for each sequence.

**Figure 1:**
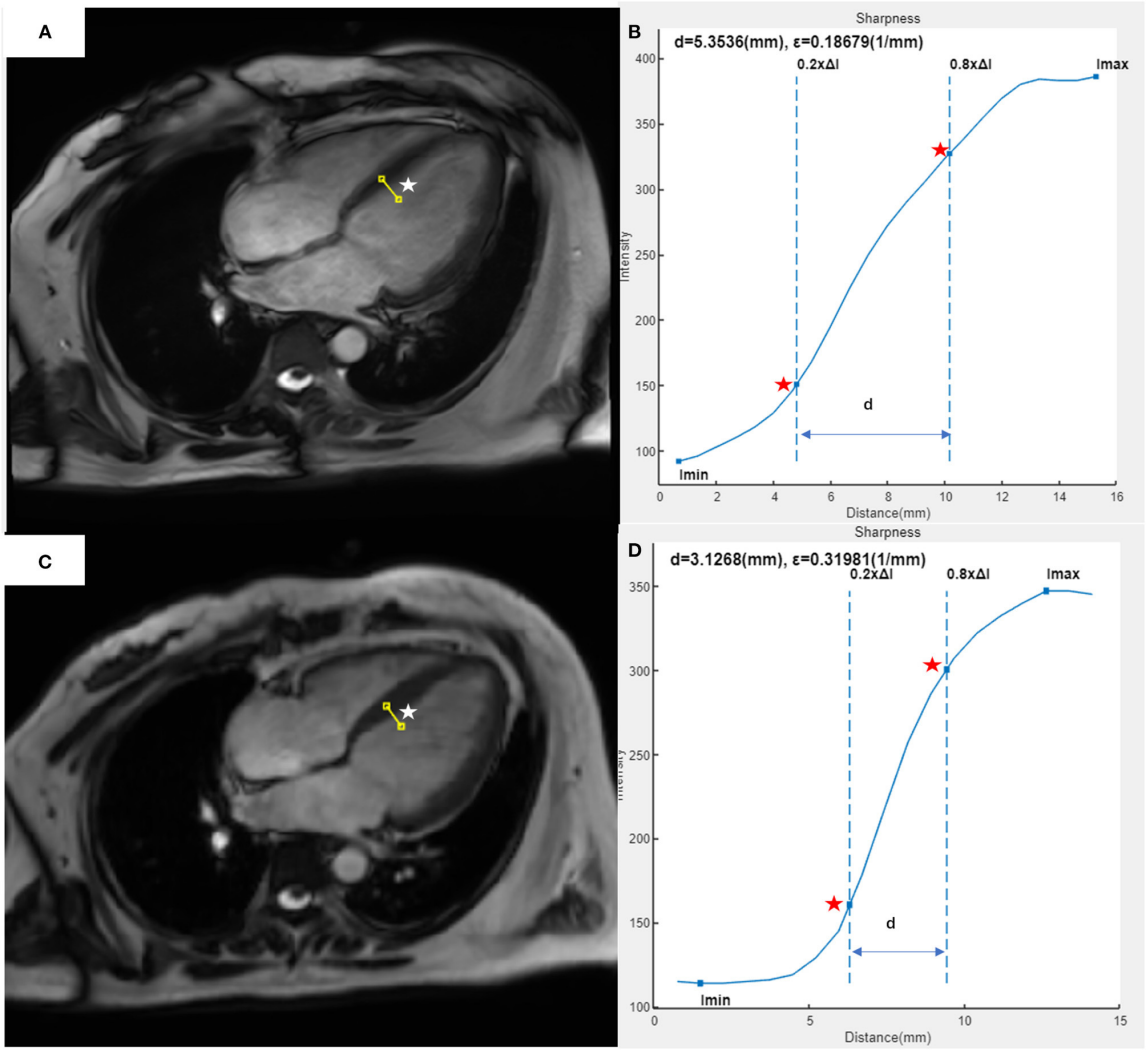
Example of edge sharpness assessment for a 37-year-old female patient with heart failure and atrial fibrillation, as performed in our study. The image of the 4CH view at end-diastole was acquired with segmented **(A)** or CSRT **(C)** bSSFP cine sequence. A line (white star) was drawn perpendicularly to the mid-cavity interventricular septum border with the left ventricular blood pool on each image. In the intensity profile (blue curve) of each line from the segmented cine **(B)** or CSRT cine **(D)**, the edge sharpness (ε, expressed in mm^−1^) was calculated as the inverse of the distance [**(D)**, expressed in mm] between the positions (red stars) corresponding to 20% and 80% of the difference between the maximum and minimum signal intensities along the line. 4CH, four-chamber; CSRT, compressed sensing real-time; bSSFP, balanced Steady-State-Free-Precession.

The edge sharpness (ε, expressed in mm^−1^) was defined as the inverse of the distance (mm) between the positions corresponding to 20% and 80% of the difference between the maximum and minimum signal intensities along the line drawn perpendicularly to the mid-cavity interventricular septum border with the LV blood pool on the end-diastole four-chamber view (17). This assessment was performed using a homemade script in MATLAB (version R2021a; The MathWorks, Natick, MA, USA) (Figure 1).

### LV Functional Quantification

**Figure 2:**
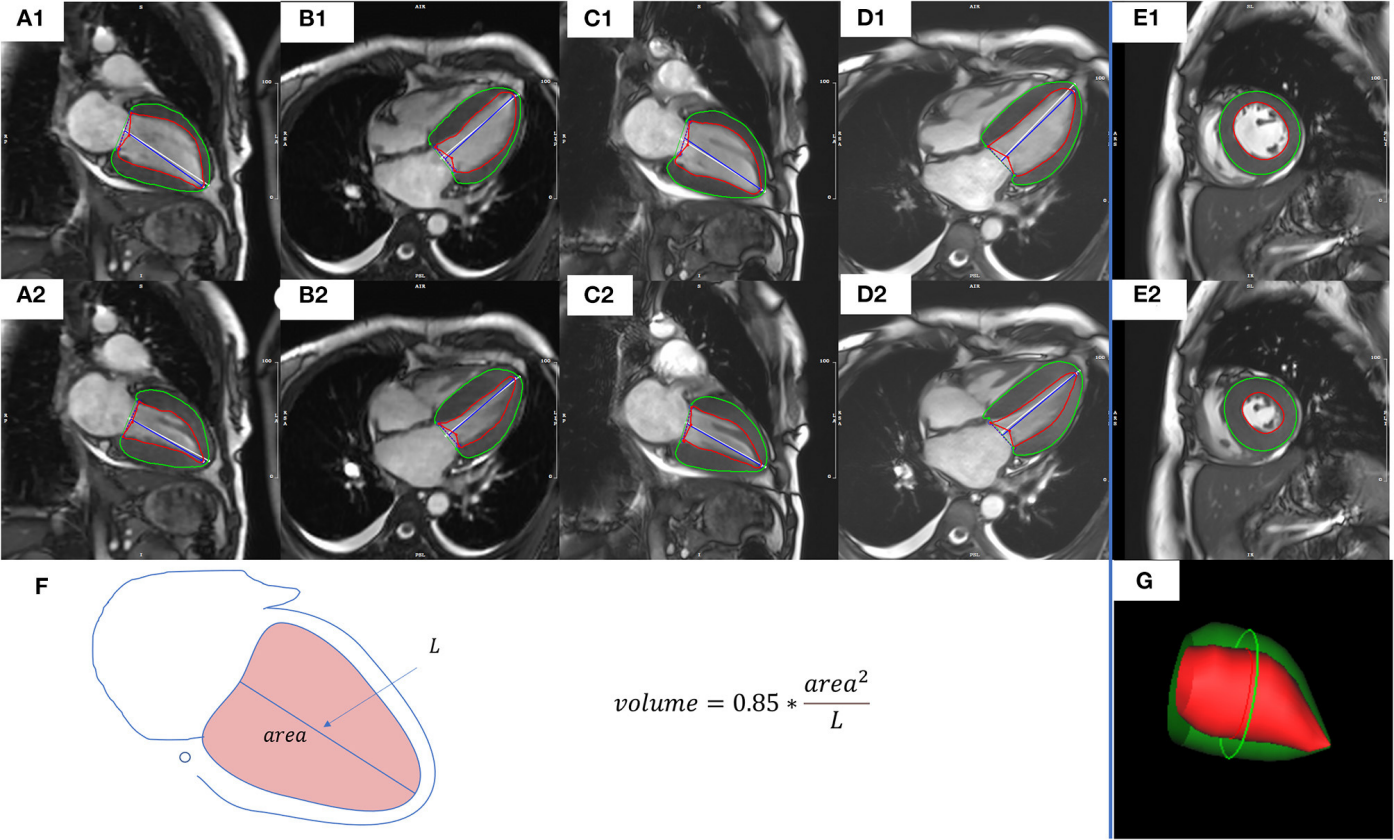
Representative images and measures of LV function from a representative study patient. CSRT2CH_AL **(A1,A2)** or CSRT4CH_AL **(B1,B2)** utilized CSRT cine combined with the 2CH or 4CH AL method. Seg2CH_AL **(C1,C2)** or Seg4CH_AL **(D1,D2)** utilized segmented cine combined with the 2CH or 4CH AL method. SegSA_Simpson **(E1,E2)** utilized segmented cine combined with SA Simpson method. The AL method is based on a rotational ellipsoid with a volume calculated using the formula: $volume=0.85\times \frac{area^{2}}{L}$ **(F)**. The ‘area' in the formula was obtained from the 2CH or 4CH view; the ‘L' was measured as the length of the line from the LV apex to the mitral valve annulus in the same view. In Simpson's rule, the LV volume is estimated as the sum of the cross-sectional area of multiple single slices multiplied by the slice thickness plus gap. The visualization of end-diastolic volume measured by Simpson's rule was shown in **(G)**. End-diastolic (upper row) and end-systolic (middle row) phases were defined manually, based on the smallest and largest LV cavities during the cardiac cycle. The endocardial contours (red) and epicardial contours (green) were drawn automatically with manual correction. CSRT2CH_AL, area-length method using two-chamber compressed sensing real time cine; CSRT4CH_AL, area-length method using four-chamber compressed sensing real time cine; Seg2CH_AL, area-length method using two-chamber segmented cine; CSRT4CH_AL, area-length method using four-chamber segmented cine; AL, area-length; SegSA_Simpson, Simpson method using short axis segmented cine, 2CH, two-chamber; 4CH, four-chamber; SA, short axis. LV, left ventricular.

Assessment of end-diastolic volume (EDV), end-systolic volume (ESV), stroke volume (SV), ejection fraction (EF), cardiac output (CO), cardiac index (CI), and LV mass (LVM) were performed using dedicated CMR postprocessing software (CVI42; Circle Cardiovascular Imaging, Inc., Calgary, Canada). For all patients with NSR, the 5 methods used for functional assessment were as follows: CSRT cine images combined with AL method in 2CH (CSRT2CH_AL) or 4CH (CSRT4CH_AL), segmented cine images combined with AL method in 2CH (Seg2CH_AL) or 4CH (Seg4CH_AL), and segmented cine images combined with Simpson's rule in SA (SegSA_Simpson) (Figure 2). The AL method is based on a rotational ellipsoid with the volume calculated using the formula: $volume=0.85\times \frac{area^{2}}{L}$. The ‘area' in the formula was obtained from the 2CH or 4CH view; the ‘L' was measured as the length of the line from the LV apex to the center of the mitral valve annulus in the same view (16, 18). In Simpson's rule, the LV volume is estimated as the sum of the cross-sectional area of multiple single slices multiplied by the slice thickness plus gap. The endo- and epicardial contours were detected automatically with manual correction. The endocardial trabeculations and papillary muscles were included in the ventricular volume. End-diastolic and end-systolic phases were defined manually, based on the smallest and largest LV cavities during the cardiac cycle (19). For all patients with AF, LV functional parameters and heart rate (HR) for every consecutive heartbeat up to 4 or 5 heartbeats were measured using CSRT2CH_AL. Then, the mean, maximum, and minimum values of each parameter from the 4~5 consecutive heartbeats were calculated as three new quantitative indices for each functional parameter. To test intra-observer variability, the functional analysis was repeated two months later by the same radiologist, for a subset of 20 randomly selected participants with NSR and all participants with AF. The inter-observer variability also was tested by another radiologist (one with 5 years of experience) in the same subset of participants.

### Statistical Analysis

Continuous variables are presented as mean ± SD, or as median (first quartile, third quartile) if not normally distributed. Categorical variables are presented as numbers (percentages). The Shapiro-Wilk test was applied to test for normally distributed data. The interobserver agreement of image quality was determined by the intraclass correlation coefficient (ICC). For both sequences, statistical effects on image quality were determined using the Wilcoxon matched-pairs signed rank test. Statistical effects on edge sharpness were determined using the paired *t*-test. For EDV, ESV, SV, EF, and LVM derived from each of the above five methods in patients with NSR or the mean, maximum, and minimum values of each EDV, ESV, SV, EF, CO, CI, HR, or LVM from the 4~5 consecutive heartbeats in patients with AF, the two-way analysis of variance (ANOVA) test with *post hoc* least significant difference (LSD) tests or Friedman test with *post hoc* LSD tests was applied for comparisons, depending on data normality. The ICC, linear regression, and Bland-Altman analysis were used to assess correlations and agreements between SegSA_Simpson and CSRT2CH_AL or CSRT4CH_AL in patients with NSR. Intra- and inter-observer reproducibility of CSRT2CH_AL and CSRT4CH_AL were assessed in twenty randomly selected patients with NSR. Intra- and inter-observer reproducibility by ICC of mean, maximum and minimum values of each functional parameter from 4~5 heartbeats were assessed in all AF patients. p values <0.05 were considered significant. All data were analyzed using IBM SPSS version 23(SPSS Inc, Chicago, IL) and Prism statistical software package (version 8.0.2, GraphPad Software, San Diego, CA, USA).

## Results

### Baseline Characteristics

Seventy-one patients successfully completed CMR scans in the present study. Demographic data are listed in Table 2. The total acquisition times (mean ± SD), including the time of voice commands and pauses between breath-holds, for the 71 subjects were 474.2 ± 72.3 s for the segmented cine sequence and 81.4 ± 5.8 s for the CSRT cine sequence (*p* < 0.001).

**Table 2:** Study population characteristics.

	**NSR Patients** **(*n =* 36)**	**AF Patients** **(*n =* 35)**
Age, yr	44.2 ± 14.5	56.9 ± 11.2
Male	24 (66.7)	25 (71.4)
BSA, m^2^	1.8 ± 0.2	1.8 ± 0.2
BMI, kg/m^2^	24.7 ± 4.3	25.6 ± 3.1
Rest heart rate, beats/min	66.3 ± 11.2	88.2 ± 20.2
**Cardiovascular disease**		
Cardiomyopathy	21 (58.3)	16 (45.7)
Myocarditis	3 (8.3)	0 (0)
Valve disease	4 (11.1)	5 (14.3)
CAD	1 (2.8)	2 (5.7)
HF	0 (0)	4 (11.4)
Other	7 (19.4)	8 (22.9)

*Values are provided as mean ± SD or n (%). BSA, body surface area; BMI, body mass index; CAD, coronary artery disease; HF, heart failure; NSR, normal sinus rhythm; AF, atrial fibrillation*.

### Image Quality

#### Semi-quantitative Analysis

**Figure 3:**
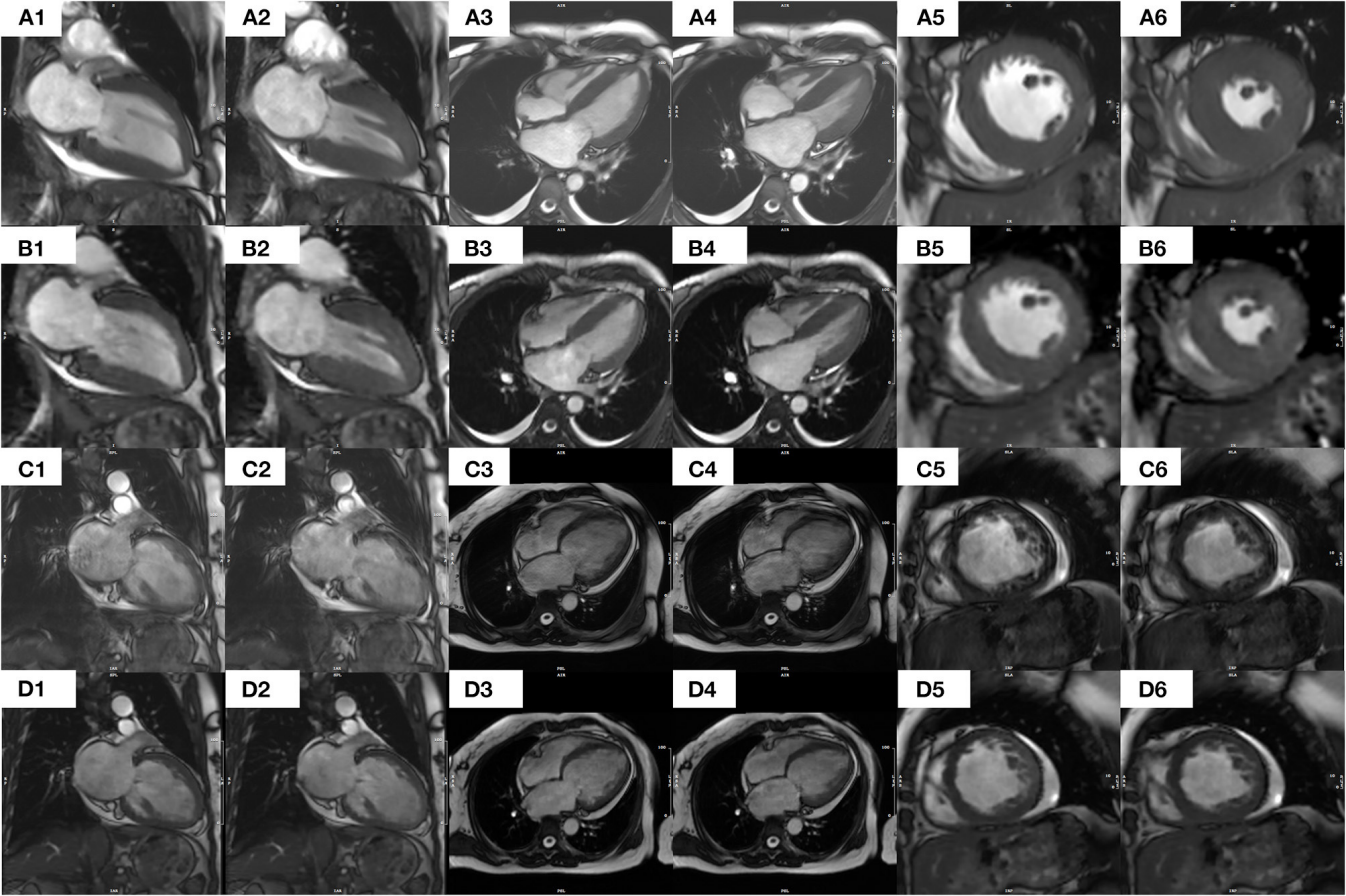
Images of segmented and CS real-time bSSFP cine sequences from a patient with NSR [**(A1–A6)** and **(B1–B6)**, respectively] and a patient with AF [**(C1–C6)** and **(D1–D6)**]. Specifically, in a 41-year-old female patient with NSR, each image from a segmented cine in end diastolic **(A1)** or end systolic **(A2)** 2CH, end diastolic **(A3)**, or end systolic **(A4)** 4CH, or end Diastolic **(A5)** or end systolic **(A6)** middle SA appears clearer and sharper than the corresponding images from a CS real-time cine [**(B1–B6)**, respectively] In contrast, in a 54-year-old female patient with AF, the images of a segmented cine appear blurrier and of lower diagnostic value. Specifically, each image from a CS real-time cine in end diastolic **(D1)** or end systolic **(D2)** 2CH, end diastolic **(D3)** or end systolic **(D4)** 4CH, or end diastolic **(D5)** or end systolic **(D6)** middle SA appears clearer and sharper than the corresponding images from a segmented cine [**(C1–C6)**, respectively]. An additional movie file shows this in more detail. CS, compressed sensing; bSSFP, balanced Steady-State-Free-Precession; AF, atrial fibrillation; NSR, normal sinus rhythm; 2CH, two-chamber; 4CH, four-chamber; SA, short axis.

In all 36 patients with NSR, the segmented cine image quality score was higher than that of the CSRT cine (3.79 ± 0.36 vs. 3.12 ± 0.39, respectively; *p* < 0.001). In contrast, in all 35 AF patients, the CSRT cine image quality was higher than that of the segmented cine (2.96 ± 0.61 vs. 1.90 ± 0.70, respectively; *p* < 0.001). In all 71 patients (including both NSR and AF), there was no significant difference in image quality between CSRT and segmented cine (3.04 ± 0.52 vs. 2.86 ± 1.10, respectively; *p* = 0.152). All images of both sequences in NSR and CSRT sequence in AF were regarded as diagnostic images, but almost half of the AF patients (17 of 35) had a segmented cine image quality score lower than 2 (nondiagnostic). Interobserver agreements for the image quality regarding segmented cine and CSRT cine from all patient data were 0.870 and 0.872 (in NSR), 0.943 and 0.934(in AF), respectively. Representative images from the two sequences in patients with NSR and AF are provided in Figure 3.

#### Edge Sharpness

In all 36 patients with NSR, the edge sharpness of the segmented cine was higher than that of the CSRT cine (0.448 ± 0.109 mm^−1^ vs. 0.363 ± 0.070 mm^−1^, respectively; *p* < 0.001). In contrast, in all 35 AF patients, the edge sharpness of the CSRT cine was higher than that of the segmented cine (0.335 ± 0.063 mm^−1^ vs. 0.284 ± 0.089 mm^−1^, respectively; *p* < 0.001). In all 71 patients (including both NSR and AF), there was no significant difference between the segmented cine and CSRT cine (0.367 ± 0.129 mm^−1^ vs. 0.349 ± 0.067 mm^−1^, respectively; *p* = 0.177).

### LV Functional Quantification

#### Patients With Normal Sinus Rhythm

**Figure 4:**
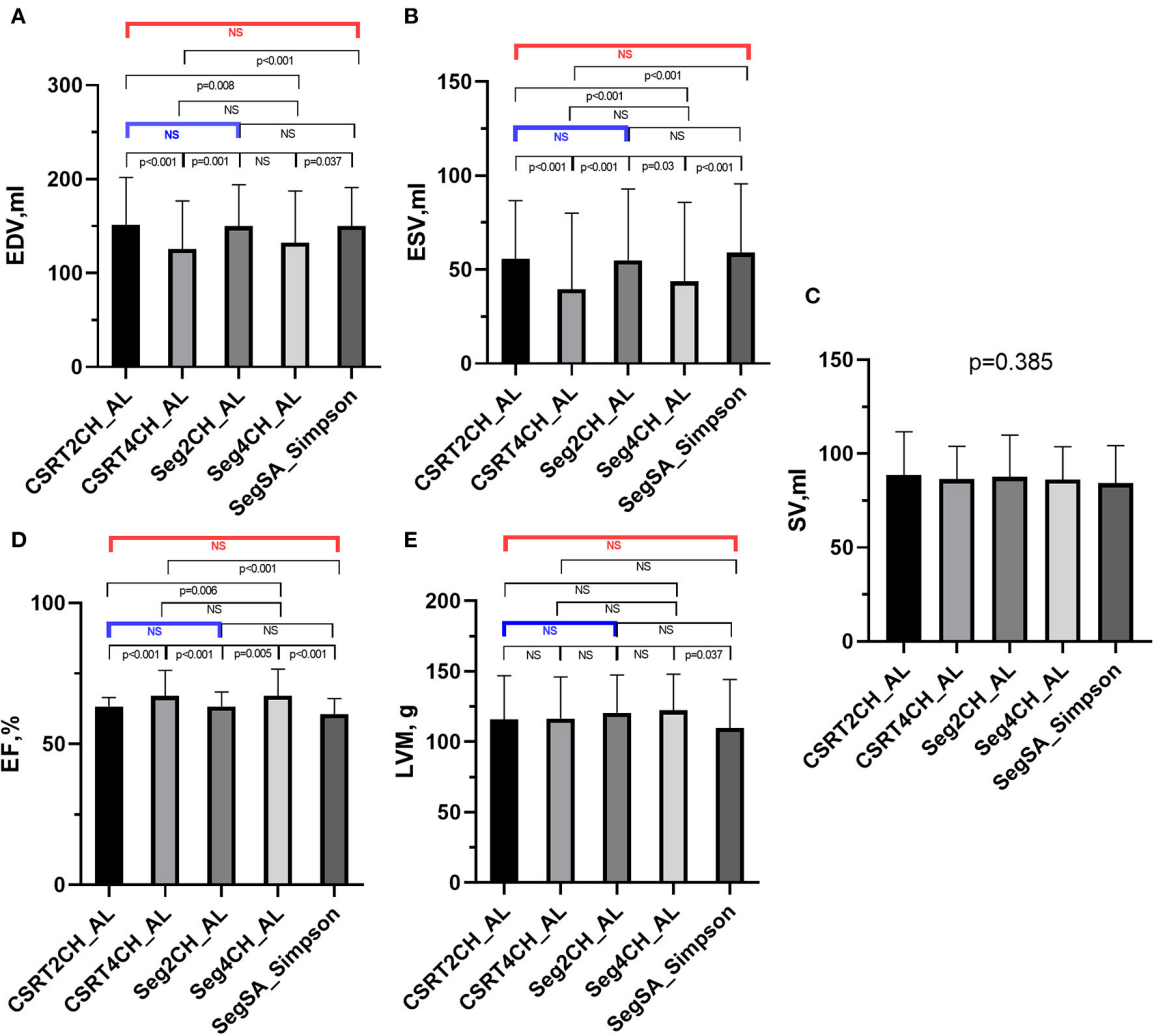
The results of multiple group comparisons and further pairwise comparisons of EDV **(A)**, ESV **(B)**, SV **(C)**, EF **(D)** or LVM **(E)** derived from 5 different methods in patients with NSR. There were significant differences in EDV, ESV, EF, and LVM but no significant difference in SV among the 5 methods. Further pairwise analyses indicated no significant differences in EDV, ESV, EF, and LVM between CSRT2CH_AL and SegSA_Simpson (red bar), and between CSRT2CH_AL and Seg2CH_AL (blue bar). There were significant differences in EDV, ESV, and EF between CSRT4CH_AL and SegSA_Simpson. CSRT2CH_AL, area-length method using two-chamber compressed sensing real time cine; CSRT4CH_AL, area-length method using four-chamber compressed sensing real time cine; Seg2CH_AL, area-length method using two-chamber segmented cine; CSRT4CH_AL, area-length method using four-chamber segmented cine; SegSA_Simpson, Simpson method using short axis segmented cine; NSR, normal sinus rhythm; EDV, end-diastolic volume; ESV, end-systolic volume; SV, stroke volume; EF, ejection fraction; LVM, left ventricular mass; NS, no significant difference.

LV functional quantitative data for patients with NSR are summarized in Table 3 and Figure 4. There were no significant differences among the 5 different methods in SV. However, significant differences were seen among the 5 methods in EDV, ESV, EF, and LVM. Further pairwise analysis indicated that both EDV and ESV derived from CSRT4CH_AL or Seg4CH_AL were significantly lower than those derived from SegSA_Simpson. EF derived from CSRT4CH_AL or Seg4CH_AL was significantly larger than that derived from SegSA_Simpson. LVM derived from Seg4CH_AL was significantly larger than that derived from SegSA_Simpson. There were no significant differences between CSRT2CH_AL and SegSA_Simpson, between Seg2CH_AL and SegSA_Simpson, between CSRT2CH_AL and Seg2CH_AL, or between CSRT4CH_AL and Seg4CH_AL for any of the LV functional measurements determined in this study. However, there were significant differences between CSRT2CH_AL and CSRT4CH_AL for EDV, ESV, and EF, though not for LVM.

**Table 3:** LV functional comparisons in patients with normal sinus rhythm.

	CSRT2CH_AL*	**CSRT4CH_AL^**#**^**	**Seg2CH_AL†**	**Seg4CH_AL∞**	**SegSA_Simpson**	***p***
EDV, mL	151.5 (120.6,202.0)	125.7 (106.7,176.9)*	150.0 (119.3,194.0)#	132.5 (109.5,187.4)*	150.2 (114.2,191.1)#**∞**	**<0.001**
ESV, mL	55.9 (38.6,86.7)	39.6 (27.5,80.0)*	54.9 (38.8,92.9)#	43.7 (28.3,85.8)*†	59.1 (40.2,95.7)#**∞**	**<0.001**
SV, mL	88.6 ± 23.1	86.4 ± 17.6	87.7 ± 22.3	86.2 ± 17.4	84.3 ± 20.0	0.385
EF, %	63.3 (47.2,66.5)	67.1 (50.5,76.1)*	63.3 (44.4,68.5)#	67.1 (48.1,76.5)*†	60.5 (42.1,66.1) **#∞**	**<0.001**
LVM, g	115.9 (89.8,147.0)	116.5 (94.1,146.1)	120.6 (92.9,147.5)	122.4 (100.9,148.1)	110.0 (91.7,144.3) **∞**	**0.033**

Values are provided as mean ± SD or median (interquartile range). Bold values are statistically significant. ^*^p <0.05 vs. CSRT2CH_AL results. ^#^p <0.05 vs. CSRT4CH_AL results. ^†^p <0.05 vs. Seg2CH_AL results. **∞**p <0.05 vs. Seg4CH_AL results.

*CSRT2CH_AL, area-length method using two-chamber compressed sensing real time cine; CSRT4CH_AL, area-length method using four-chamber compressed sensing real time cine; Seg2CH_AL, area-length method using two-chamber segmented cine; CSRT4CH_AL, area-length method using four-chamber segmented cine; SegSA_Simpson, Simpson method using short axis segmented cine; EDV, end-diastolic volume; ESV, end-systolic volume; SV, stroke volume; EF, ejection fraction; LVM, left ventricular mass; LV, left ventricular*.

**Figure 5:**
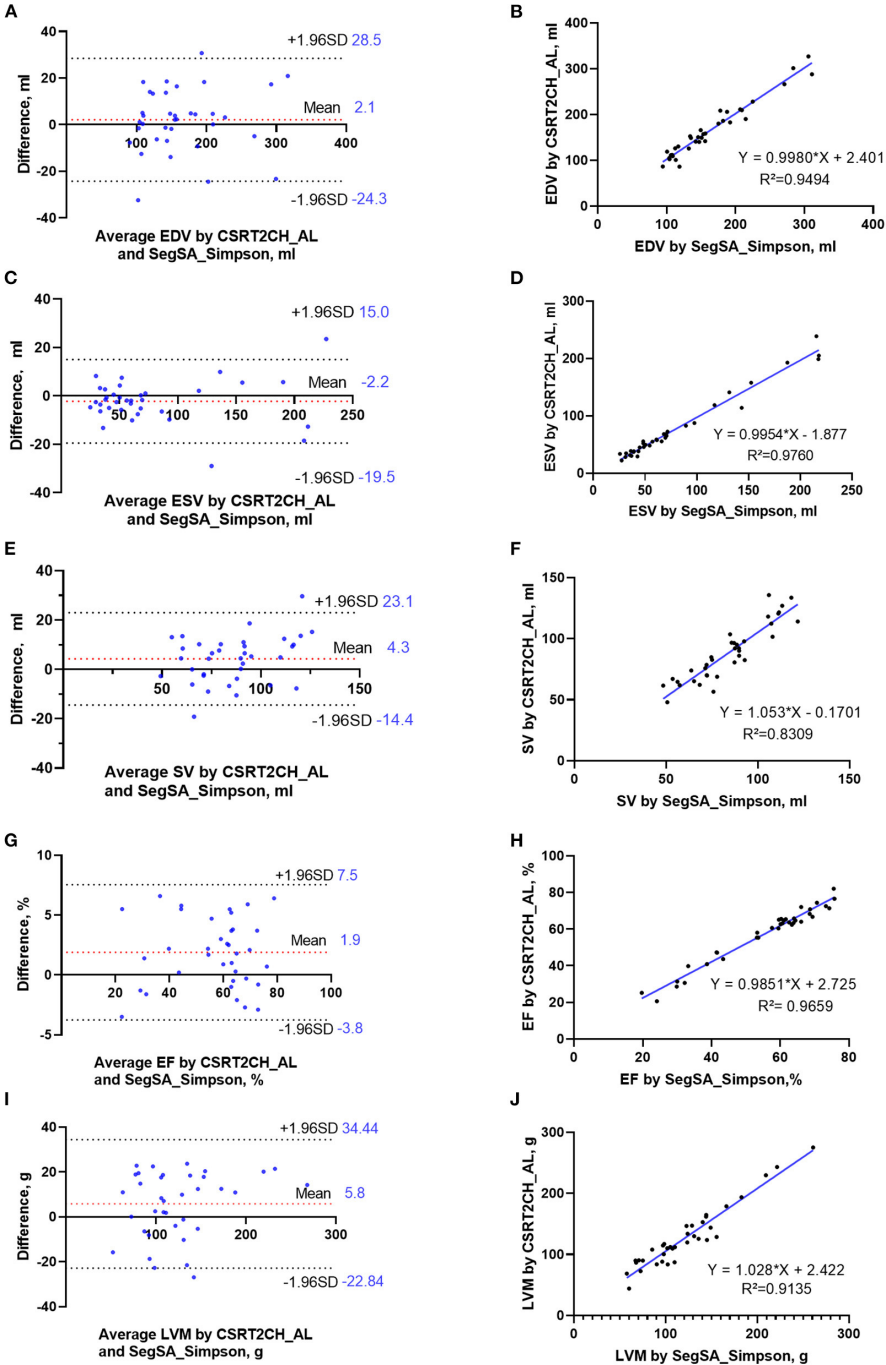
Bland-Altman plots and linear regression trendlines for LV functional measurements by CSRT2CH_AL and SegSA_Simpson in patients with NSR. The diagrams in the left column represent Bland-Altman plots for EDV **(A)**, ESV **(C)**, SV **(E)**, EF **(G)**, and LVM **(I)**. The red-dashed lines indicate the difference between two methods; the black-dashed lines indicate the 95% limit agreement interval. On the diagrams in the right column, linear regression analysis indicates strong correlations in EDV **(B)**, ESV **(D)**, SV **(F)**, EF **(H)** and LVM **(J)** between CSRT2CH_AL and SegSA_Simpson. CSRT2CH_AL, area-length method using two-chamber compressed sensing real-time cine; SegSA_Simpson, Simpson method using short axis segmented cine; NSR, normal sinus rhythm; EDV, end-diastolic volume; ESV, end-systolic volume; SV, stroke volume; EF, ejection fraction; LVM, left ventricular mass; LV, left ventricular.

According to the ICC analysis, there was good agreement in all parameters between CSRT2CH_AL and SegSA_Simpson. Similarly, good agreement was found in all parameters except for SV (ICC: 0.734, *p* < 0.001) between CSRT4CH_AL and SegSA_Simpson. The ICC value for each LV functional parameter compared between CSRT2CH_AL and SegSA_Simpson was better than that between CSRT4CH_AL and SegSA_Simpson (Table 4). In patients with NSR, good agreement also was obtained for LV functional quantitative data when comparing CSRT2CH_AL and SegSA_Simpson, as assessed by Bland-Altman and linear regression analyses (Figure 5).

**Table 4:** Intraclass correlation coefficient analysis of functional measurements in patients with NSR and AF.

	**NSR**						**AF**					
	**ICC between** **CSRT2CH_AL** **and SegSA_Simpson**	**ICC between CSRT4CH_AL** **and SegSA_Simpson**	**ICC - CSRT2CH_AL**		**ICC – CSRT4CH_AL**		**ICC - Mean**		**ICC -Maximum**		**ICC- Minimum**	
			**Intra**	**Inter**	**Intra**	**Inter**	**Intra**	**Inter**	**Intra**	**Inter**	**Intra**	**Inter**
EDV,mL	0.974	0.966	0.993	0.986	0.986	0.988	0.997	0.997	0.994	0.990	0.996	0.994
ESV,mL	0.988	0.971	0.996	0.992	0.992	0.992	0.997	0.996	0.996	0.994	0.995	0.994
SV, mL	0.902	0.734	0.976	0.933	0.845	0.875	0.992	0.967	0.964	0.929	0.985	0.970
EF, %	0.983	0.931	0.987	0.985	0.964	0.961	0.994	0.987	0.973	0.956	0.977	0.974
LVM, g	0.953	0.948	0.990	0.967	0.975	0.964	0.986	0.977	0.976	0.956	0.975	0.964

*ICC analysis was used to assess the agreements for LV functional quantitative data between CSRT2CH_AL and SegSA_Simpson, and between CSRT4CH_AL and SegSA_Simpson in patients with NSR, and inter- and intra-observer agreements for LV functional quantitative data in patients with NSR and AF.*

*CSRT2CH_AL, area-length method using two-chamber compressed sensing real time cine; CSRT4CH_AL, area-length method using four-chamber compressed sensing real time cine; SegSA_Simpson, Simpson method using short axis segmented cine; EDV, end-diastolic volume; ESV, end-systolic volume; SV, stroke volume; EF, ejection fraction; LVM, left ventricular mass; NSR, normal sinus rhythm; AF, atrial fibrillation; LV, left ventricular; ICC, intraclass correlation coefficient; Inter, inter-observer; Intra, intra-observer*.

#### Patients With Sustained Atrial Fibrillation

The mean heartrate in all 35 AF patients was 88.2 ± 20.2 bpm. We successfully measured LV functional parameters for every heartbeat up to 4 or 5 heartbeats in patients with AF (4 heartbeats for three patients with average heart rate <60 beats/min; 5 heartbeats for the remaining 32 patients) using the CSRT2CH_AL method. There were significant differences among the mean, maximum, and minimum values of each parameter (EDV/ESV/SV/EF/CO/CI/LVM/HR) in the acquired 4~5 consecutive heartbeats. The three new quantitative indices for each functional parameter in patients with AF are summarized in Table 5.

**Table 5:** LV functional comparisons in patients with atrial fibrillation.

	Mean*	**Maximum#**	**Minimum**	***p***
EDV, mL	212.3 ± 111.7#	231.2 ± 114.9*	191.8 ± 111.6*#	**<0.001**
ESV, mL	127.9 (57.0, 212.4)#	141.8 (69.1, 232.8)*	109.3 (46.9, 195.8*#)	**<0.001**
SV, mL	62.3 (48.1,81.9)#	88.6 (67.7, 111.2)*	35.1 (25.2, 52.0)*#	**<0.001**
EF, %	36.4 ± 14.9#	45.5 ± 15.0*	27.0 ± 15.4*#	**<0.001**
CO, L/min	4.9 (4.1, 6.8)#	7.5 (5.8,9.1)*	3.3 (2.3, 4.4)*#	**<0.001**
CI, L/min/m^2^	2.8 (2.3, 3.6)#	4.1 (3.5, 4.8)*	1.9 (1.3, 2.4)*#	**<0.001**
LVM, g	116.8 ± 42.2#	132.1 ± 48.1*	104.1 ± 40.0*#	**<0.001**
HR, beats/min	88.2 ± 20.2#	111.0 ± 33.5*	68.0 ± 16.6*#	**<0.001**

Values are provided as mean ± SD or median (interquartile range). Bold values are statistically significant. *p <0.05 vs. Mean results. #p <0.05 vs. Maximum results.

*EDV, end-diastolic volume; ESV, end-systolic volume; SV, stroke volume; EF, ejection fraction; CO, cardiac output; CI, cardiac index; HR, heart rate; LVM, left ventricular mass; LV, left ventricular*.

#### Intra- and Inter-observer Variability

The ICCs of quantitative LV functional data in patients with NSR and AF are summarized in Table 4. In patients with NSR, intra-observer and inter-observer variabilities were small, as evidenced by ICC values ranging from 0.976 to 0.996 and 0.933 to 0.992 (respectively) for measurements by CSRT2CH_AL, and from 0.845 to 0.992 and 0.875 to 0.992 for measurements by CSRT4CH_AL. In patients with AF, intra-observer and inter-observer variabilities also were small, as evidenced by ICC values ranging of 0.986 to 0.997 and 0.967 to 0.997 (respectively) for mean values, 0.964 to 0.996 and 0.929 to 0.994 for maximum values, and 0.977 to 0.996 and 0.964 to 0.994 for minimum values.

## Discussion

This prospective study was based on a 71-patient cohort including patients with various cardiac disease with NSR or AF. The results demonstrated that a CSRT cine CMR yielded slightly worse scores for image quality and edge sharpness, compared to a standard cine CMR, in patients with NSR. In contrast, in patients with AF, the image quality and edge sharpness of a CSRT cine CMR both were higher than those of a segmented cine. Compared to a segmented cine using Simpson's method, a CSRT bSSFP sequence using the AL method in 2CH was shown to be an accurate method for measuring global LV parameters, including EDV, ESV, SV, EF, and LVM, in patients with NSR. In patients with AF, the CSRT2CH_AL method provided significantly different results among mean, maximum, and minimum values for each LV functional parameter over multiple consecutive heartbeats, while exhibiting excellent inter- or intra-observer reproducibility. In addition, the CS real-time cine acquisition was more timesaving than multi-breath-hold segmented cine acquisition, and may be particularly beneficial for ill patients who cannot hold their breath or tolerate long-duration scans.

In the segmented cine sequence, using ECG gating for data synchronization, the raw data of the integrated k-space is acquired segmentally in relatively narrow time windows (high temporal resolution) for reconstruction of multiple-image frames (cardiac phases) representing the cardiac cycle. This process is repeated over multiple heartbeats to fill the entire k-space for each cardiac phase (20, 21). This approach works well in patients with a regular rhythm. However, when arrythmia occurs, the status of the moving heart in abnormal heartbeat differs from that in other heartbeats. Since data synchronization does not work well in k-space segmentation, the acquisition data of a segmented cine maybe incorrect and the images may appear to be blurred. On the other hand, because the number of reconstructed phases of one cardiac cycle in each acquired slice is the same no matter how long the RR interval is, the order of end-systolic and/or end-diastolic phase in different slices maybe different in cases with arrhythmia. As a result, Simpson's method using multiple slices for disk summation will (in theory) overestimate ESV and/or underestimate EDV, and finally underestimate SV and EF. As a result, real-time acquisition is essential to characterize LV function in patients with severe arrhythmia, such as AF (9). Usually, the spatial and temporal resolution of real-time acquisition, such as non-Cartesian sampling or usage of ultrahigh sense factor, is seriously restricted due to the MR technical challenges. Over the last decade, the CSRT sequence has emerged and has proved to be an alternative tool for cardiac functional assessment (11, 12). Similar spatial and temporal image resolutions are maintained by using an under-sampling technique and an iterative reconstruction algorithm, compared to those obtained with a routine segmented cine (13). CS real-time bSSFP cine imaging has been shown to be suitable for assessing ventricular interdependence or cardiac function in patients with irregular rhythms or who are unable to hold their breath (22). Several previous studies in non-selected patients have confirmed that CS real-time cine imaging is an alternative tool for cardiac functional assessment compared to segmented multi-breath-hold bSSFP (13, 17, 23). In the present study, the image quality and edge sharpness of CS real-time cine images were superior to those of routine segmented cine images in patients with AF. These results are consistent with the recent study by Longère et al., which demonstrated a dramatic drop in arrhythmia-related artifacts and a significant improvement of subjective and objective image quality when using a CS real-time sequence in patients suffering from heart rhythm disorders (13).

The area-length method is well known in cardiac function analysis and provides a quick and simple evaluation method for LV systolic function; this approach is highly accepted in echocardiography and angiography (16). As early as 1980, Wyatt et al. demonstrated, in an *in vitro* study of cross-sectional echocardiography, that the volume of the symmetric left ventricle can be quantified reliably using an AL method (14). Subsequently, Hergan et al. demonstrated that the AL method may provide a quick overview of LV function in healthy volunteers, although this approach should not be used for right ventricular functional assessment (5). Huttin et al. reported the use of an AL method to provide EF values closer to transthoracic echocardiography measurements in patients with acute myocardial infarction (24). To our knowledge, the present work is the first to assess LV systolic function by CSRT cine combined with an AL method in patients with various cardiac diseases, including a comparison to reference cases; we hope that this technique can be employed in patients with AF. Given that the 2CH and 4CH views were not acquired simultaneously in CMR, the biplane method using the two long-axis views was not employed in the present study. However, the AL method using a single plane is based on ellipsoidal geometric assumptions and may lead to inaccurate volumetric or functional estimation, especially in patients with asymmetrical geometry or regional dysfunction. Therefore, we intentionally excluded patients with asymmetric left ventricles and/or non-uniform myocardial contraction.

In patients with NSR, our study demonstrated that all LV functional parameters obtained by CSRT2CH_AL were equivalent to those obtained by SegSA_Simpson. However, significant systematic differences were found in the values of EDV, ESV, and EF when comparing between the CSRT4CH_AL and Simpson's methods. In addition, all of the CSRT2CH_AL-derived parameters exhibited stronger correlations to those obtained by SegSA_Simpson compared to those generated by CSRT4CH_AL. Although no significant difference was found in SV between the CSRT4CH_AL and Simpson's methods, the agreement between the SV values obtained by these two approaches was relatively poor. This observation may reflect the targeting method of the 4CH plane in the CMR scanning practice: according to the standardized protocols of the Society for Cardiovascular Magnetic Resonance (SCMR), the 4CH long-axis view is prescribed from the 2CH long-axis view through the apex and center of the mitral and tricuspid valves (22). Subsequently, the plane of the 4CH view usually was tilted for crossing the center of the tricuspid valves by cross-checking on the basal short axis view. As a result, the LV may not have been bisected by the 4 CH plane in our study. Additionally, the area of the LV in the 4 CH view usually is smaller than that of the median section of the LV.

In clinical practice, LV EF, which is still the most widely used parameter of LV systolic function, has an important prognostic value for multiple kinds of cardiac disorders, including AF (6). In patients with AF, evaluation of LV function may be complicated by irregular rhythm and elevated heart rate, and as a rule LV function should be assessed across multiple consecutive cardiac cycles (25). Furthermore, AF is one of the major causes of tachycardia-induced cardiomyopathy, which is a condition in which atrial or ventricular tachyarrhythmias or frequent ventricular ectopy result in LV dysfunction, leading to systolic heart failure. After the correction of the arrhythmia or the control of the rhythm, LV systolic dysfunction can recover completely or partially (6). Thus, the mean and maximum EF, SV, or other functional parameter in multiple consecutive beats may be of clinical value. Because of the irregularity in the heart rate in AF status, the EF has variance during different heart beats. The measurements of beat-to-beat variation may contribute to revealing the underlying cause of AF and facilitate the earlier initiation of medical care (26). David Ouyang et al. reported video-based artificial intelligence for beat-to-beat assessment of cardiac function in 2020 in the journal Nature (27). And their work showed variation in beat-to-beat model interpretation in echocardiogram videos of patients with arrhythmias and ectopy. We observed significant differences among the mean, maximum, and minimum values of every LV functional parameter derived via the CSRT2CH_AL method over 4~5 consecutive heartbeats in patients with AF. Nonetheless, all of the results exhibited good inter- or intra-observer reproducibility. These differences suggest that one-beat segmented cines are not suitable for LV functional assessment in AF patients. In the future, the mean, maximum, or minimum values of each functional parameter in consecutive heartbeats will need to be investigated further in patients with AF.

### Limitations

Our study had some limitations. Firstly, because there is no consensus on systolic measurements in AF, we did not find a “gold standard” to verify quantitative functional measurements in patients with AF in our study. Secondly, image edge sharpness was not assessed for every phase of the two cines, given the large number of the phases and the time-consuming nature of the process. The endocardial border-blurring in the routine segmented cine images appear to be more serious and to occur more frequently in phases during cardiac systolic periods in patients with AF. Thirdly, we only calculated functional parameters for 4~5 consecutive heartbeats, given that our study did not use CSRT cine sequences of longer duration. In the future, along with the faster reconstruction and the larger storage space, the acquisition of longer-duration CSRT cine sequences would be more useful for beat-to-beat analysis of LV function in patients with AF. Lastly, we discarded the first 10 phases of each CSRT cine when performing qualitative and quantitative analyses, given our lack of dummy scans. In the future, the sequence designer can provide a choice for user to define a dummy-scan duration of several hundreds of milliseconds or of a number of heartbeats.

## Conclusion

The CSRT cine sequence provided improved image quality and edge sharpness in patients with AF. This approach also permitted accurate and convenient assessment of the LV systolic function by the two-chamber area-length method in patients with NSR. Furthermore, the CSRT cine imaging combined with two-chamber area-length method facilitated the determination of LV functional parameters in consecutive heartbeats with great inter- or intra-observer reproducibility for beat-to-beat analysis of LV function in patients with AF.

## Data Availability Statement

The datasets used or analyzed during the current study are not publicly available due to regulatory restrictions. Data that support the findings of this study are available from the corresponding author on reasonable request.

## Ethics Statement

The studies involving human participants were reviewed and approved by Ethics Committee of Fuwai Hospital. The patients provided their written informed consent to participate in this study.

## Author Contributions

GY: design of the work, data acquisition, the analysis, interpretation of data, and drafting of the manuscript. CC: design of the work, data acquisition, analysis and interpretation of data, and revision of the manuscript. JA: design of the work and revision of the manuscript. KZ: statistical analysis and revision of the manuscript. KY, XC, and ML: analysis and interpretation of data and revision of the manuscript. SL: data acquisition and statistical analysis. XY: data acquisition and MATLAB script compiling. JW, ZD, SY, and JH: data acquisition. SZ: design of the work and revision of the manuscript. All authors read and approved the final manuscript.

## Funding

The study was supported by National Key Research and Development Program of China under Award Number 2021YFF0501400 and Key project of National Natural Science Foundation of China under Award Number 81930044.

## Conflict of Interest

JA was employed by Siemens Shenzhen Magnetic Resonance Ltd. The remaining authors declare that the research was conducted in the absence of any commercial or financial relationships that could be construed as a potential conflict of interest.

## Publisher's Note

All claims expressed in this article are solely those of the authors and do not necessarily represent those of their affiliated organizations, or those of the publisher, the editors and the reviewers. Any product that may be evaluated in this article, or claim that may be made by its manufacturer, is not guaranteed or endorsed by the publisher.
